# Metabolomic Analysis on the Petal of ‘Chen Xi’ Rose with Light-Induced Color Changes

**DOI:** 10.3390/plants10102065

**Published:** 2021-09-30

**Authors:** Mengyue Su, Rebecca Njeri Damaris, Zhengrong Hu, Pingfang Yang, Jiao Deng

**Affiliations:** 1 State Key Laboratory of Biocatalysis and Enzyme Engineering, School of Life Sciences, Hubei University, Wuhan 430026, China; 201911110711029@stu.hubu.edu.cn (M.S.); njerirebecca09@gmail.com (R.N.D.); huzhengrong1001@hubu.edu.cn (Z.H.); 2 Research Center of Buckwheat Industry Technology, School of Life Sciences, Guizhou Normal University, Guiyang 550001, China

**Keywords:** rose flower, alteration color, flavonoid metabolites, anthocyanins, light induction

## Abstract

Flower color is one of the most prominent traits of rose flowers and determines their ornamental value. The color of the “Chen Xi” rose can change from yellow to red during flower blooming. In the present study, the flavonoid metabolites were investigated by the UPLC-ESI-MS/MS from the petals of four successive flower development stages under natural conditions. In total, 176 flavonoid components, including 49 flavones, 59 flavonols, 12 flavanones, 3 isoflavones, 12 anthocyanins, and 41 proanthocyanidins were identified, with some of them being detected for the first time in this study. Additionally, there were 56 compounds that showed differences among comparison groups, mainly being enriched in pathways of isoflavone, flavonoid, flavone, flavonol, phenylpropanoids, and anthocyanin. Among them, it is anthocyanins that allow the rose flower to turn red when exposed to sunlight. To verify this result, compounds from rose petal with shading treatment (S2D) was also detected but could be clearly separated from the four samples under light by clustering and principal component analyses (PCA). Consistent with low anthocyanins accumulation, the flower with shading could not turn red. Moreover, it provides a foundation for further research on the light-induced color modification of flower.

## 1. Introduction

The rose (*Rosa* sp.), belonging to the *Rosaceae* family, is one of the most popular and widely planted ornamental plants worldwide. It is also one of the top ten flowers in China, which is honored as the queen of flowers and has a long history of cultivation in China [1]. Roses are utilized as cut flowers, potted plants, and garden ornamental plants [2,3]. Due to their rich bioactive components, rose flowers are extensively used in food, drugs, cosmetics, and pharmaceutics [4,5,6,7]. Rose flowers are charming and colorful, including red, pink, orange, yellow, white, variegated, as well as alternating colors during flowering. Additionally, the blue rose flower has been cultivated through the molecular breeding method [8]. The color of the rose flower determines its ornamental and commercial value and creating novel flower colors is one of the main objectives for breeders.

It is known to all that plant color is determined by pigments, and there are four kinds of natural pigments that include flavonoids, carotenoids, chlorophyll, and alkaloids [9], which give color to plant flowers, leaves, vegetables, and fruits. Osterc [9] detected several phenolic compounds from rose petals of eight cultivars at four developmental stages, including five anthocyanins, which were regarded as the major factor for visual attributes of rose flower, and suggested that the total content of anthocyanin and quercetin (both belonging to flavonoids) continued accumulating from the bud stage until fully open flowers, then decreased in senescent ones. Previous studies indicated that in rose flowers, anthocyanins, determine the red color, carotenoids impart yellow color, while pink and orange colors are endowed by a combination of both anthocyanins and carotenoids, and nearly no pigments were detected in white flowers [10]. Lee et al. [11] identified two anthocyanins, cyanidin 3,5-di-O-glucoside and pelargonidin 3,5-di-O-glucoside in red rose flowers, with the former occupying 85% of total anthocyanins. Wan et al. [3] found nineteen flavonols and sixteen carotenoids in yellow petals of Rosa ‘Sun City’ cultivar, then they also detected four anthocyanins, 20 flavonols, and 10 carotenoids in the petals of six Rosa cultivars with yellow, pink, and orange color [10]. Combined with RNA-sequencing and metabolite analysis, Huang et al. [12] revealed the mechanism of flower color change in rose mutants. The results indicated that the expression levels of the differentially expressed genes enriched in the anthocyanin pathway, e.g., chalcone synthase (*CHS*), chalcone isomerase (*CHI*), dihydroflavonol reductase (*DFR*), and leucoanthocyanidin dioxygenase (*LDOX*) in pink flowers were significantly higher than in white flowers, which are consistent with the accumulation of anthocyanin in flowers of the two rose cultivars. By contrast, the expression of flavonol synthase (*FLS*) in the white rose flower was higher than that in red flowers, resulting in the accumulation of more flavonols. Therefore, these findings suggested that competition between anthocyanin and flavonol biosynthesis is a primary cause of rose flower color variation [12].

Light affects anthocyanin biosynthesis: for example, the anthocyanin pigmentation of some fruits’ skin including red pears [13], apples [14], and grapes [15] is induced by light. In this study, we chose one rose cultivar whose flower color changed from yellow to red during development. Before the flower opens and the petal emerges from the calyx, the flower petals are yellow with an edge which, when exposed to the sun, become red, and after the flower totally opens and the petals are fully stretched, the red color intensifies with increased exposure to the sunlight. As the flower ages, the color of the petals slowly faded to white and wither at the end. However, when the flower fully opens and is moved to the shade, the color of the yellow petals persists and does not change to red and directly fades to white as it withers. To detect if the color change is induced by light, flavonoid metabolites of rose flowers at four developmental stages under natural conditions as well as one stage flower under shading treatment were determined. The results showed the dynamic change of flavonoid metabolites during the process of color change with the flower development and lay a theoretical foundation for further understanding of the mechanism underlying the light-induced rose flower pigmentation and ultimately facilitate breeding of rose cultivars with a novel flower color.

## 2. Results

### 2.1. Light-Induced Color Changes of Rose Petals

**Figure 1:**
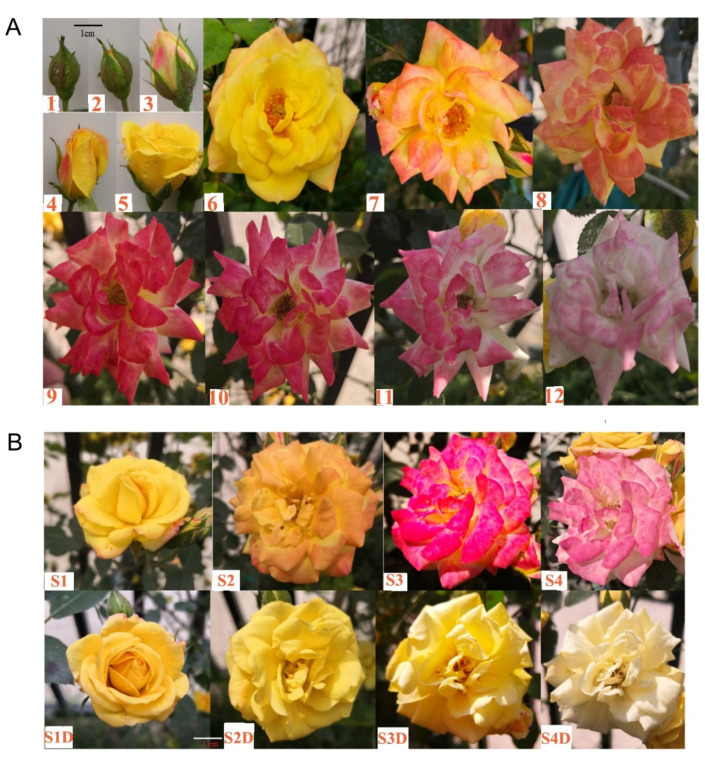
Changes in flower colors of the variety of ‘Chen Xi’ rose. (**A**) The different developmental stages of ‘Chen Xi’ rose flower from bud stage to flower blooming, and the flower color changed from yellow to red, and faded to white as it withers ((**1**–**12**) represent the order of the development stages of ‘Chen Xi’ flower). (**B**) ‘Chen Xi’ flowers at four blooming stages under natural sunlight (**S1**–**S4**) and shading (**S1D**–**S4D**).

The ‘Chen Xi’ variety of rose has a very beautiful flower showing color changes during the blooming, which contributes a lot to its ornamental value. At the bud stage, sepals show a little red color in the green background and cover the whole flower (Figure 1A). After the sepals opened, petals on the surface showed some red color with a yellow background. When the flower was just fully opened, the petals were in yellow with a tiny amount of red at the edge. Along with its development and blooming, the red is gradually increased, and then both red and yellow were gradually faded with the wilting of petals (Figure 1A). Based on the changing style of the petal’s color, we suspected that the formation of the red color is light-inducible. To verify this hypothesis, two different light treatments were carried out on the newly bloomed flowers in May 2020, natural sunlight (control group) and shading by being wrapped in a black opaque paper bag (Figure 1B). Rose flower petals at four developmental stages (named as S1, S2, S3 and S4) were observed to compare the difference of color changes between the two light treatments. At the S1 stage corresponding to the newly opened flower (Figure 1(A5)), flowers under both treatments were mainly yellow; at the S2 stage, the flower is mainly yellow with some light red (Figure 1(A7),B) under natural light, while it stayed as yellow under darkness (Figure 1); at the S3 stage, the color was deep red with fading yellow under light (Figure 1(A9),B), and flowers in the dark showed only fading yellow; at the S4 stage, the yellow was totally faded under both treatments, while only the flowers with light still showed red, although it was fading as well (Figure 1(A11),B). Altogether, it could be concluded that the changes in the red color are light dependent, but the accumulation and fading of yellow is not.

### 2.2. Metabolic Profiling of Rose Petals at Different Development Stages

**Figure 2:**
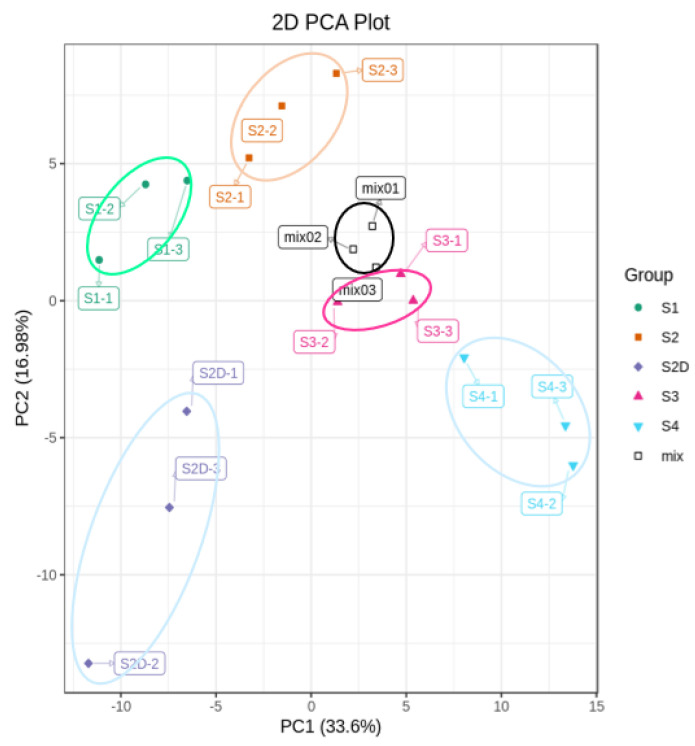
Principal component analysis (PCA) of flavonoid metabolites profiles analysis.

To explore the mechanism underlying this light-induced color change, the flavonoid metabolites of the rose petals from four different development stages and petals at the S2D stage under shading treatment (Figure 1B) were investigated by UPLC-ESI-MS/MS. A total of 176 flavonoid components were detected, including 49 flavones, 59 flavonols, 12 flavanones, 3 isoflavones, 12 anthocyanins, and 41 proanthocyanidins (Table S1), and HPLC chromatograms of these flavonoid metabolites from rose petal samples were shown in Figure S1. Principal components analysis (PCA) was carried out to assess the repeatability among three biological repeats and the variation among different samples. In the present study, two principal components PC1 and PC2 were extracted, which contributed 33.6% and 16.9%, respectively. The mix represents a sample for quality control, and PCA shows separation of the different samples and good repeatability (Figure 2). Additionally, samples of S2D could be separated from other samples by PCA (Figure 2).

**Figure 3:**
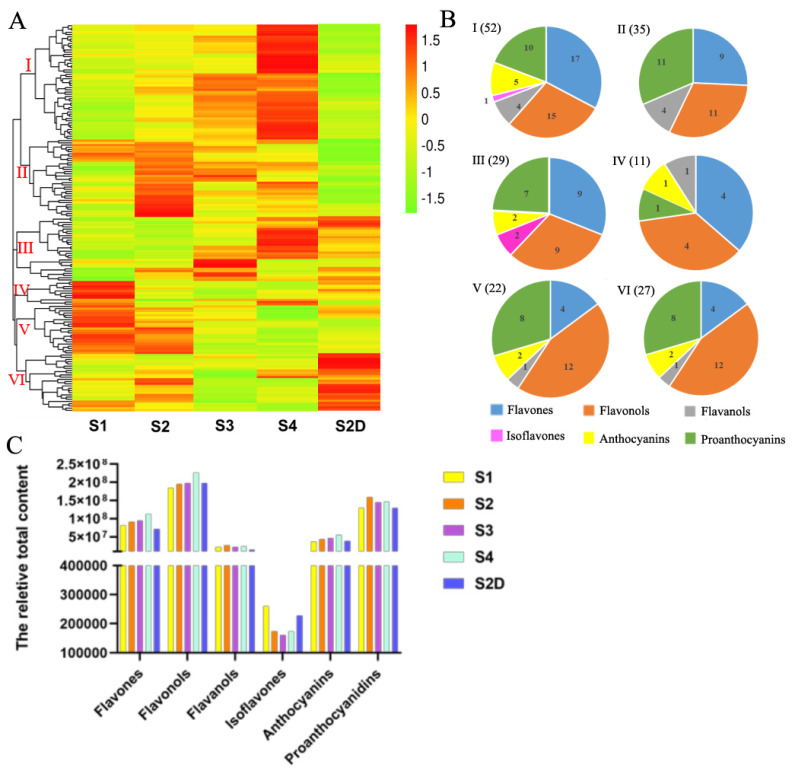
Flavonoid metabolites profile analysis. (**A**) Clustering heat map of all flavonoid metabolites, and (**B**) pie charts of the metabolites in each group. (**C**) The relative content of six types of flavonoid compounds, the content value was calculated by the peak area of each metabolite.

All the metabolites are illustrated by a heatmap, and six groups were classified based on the content of metabolites in different samples (Figure 3A). Metabolites in Group I, which had the highest number of compounds (52), mainly composed of flavones, flavonols and proanthocyanidins, had lower content in the petals of S1, then most of them increased along with the growth of the rose flower development, and there was no significant difference between S1 and S2D petals, but most of the metabolites were higher in S2 than S2D petals, while most metabolites in group II, including flavones, proanthocyanidins, flavanols, and flavonols, had the lowest content in the shading treatment (in S2D petals) but had the highest content in S2 petals. In group III, some metabolites exhibited no difference between S1 and S2 petals, then slowly increased in S3 and accumulated more in S4 petals, while some were lower in S1 petals, increased in S2 and S3, then sharply down regulated in S4 petals. Most of the metabolites in this group had higher content in petals S2D than that in S1 and S2 petals. This group also mainly contained flavones, flavonols and proanthocyanidins. Group IV had the lowest number of members, including four flavones, four flavonols, one flavanol, one anthocyanin and one proanthocyanin, almost all of them showed the highest content in S1 petals, and decreased rapidly in S2, and kept declining in S3 and S4 petals, but only several members exhibited up-regulation in S4. The majority of metabolites in the S2D of this group had slightly lower content than those in S1. In group V, flavonols and proanthocyanidins were the main members, and they kept a high content in the first two stages petals but showed a continued descent in petals of S3 and S4 petals, with most metabolites being down-regulated in S2D petals. Unlike other groups, most of the flavonoid metabolites in group VI, which included 12 flavones, 8 proanthocyanidins, 4 flavonols, 2 anthocyanins, and 1 flavanol, had the highest content in S2D, while they had a lower expression in S1, S3, and S4 flowers (Figure 3A). Specifically, eriodictyol (dihydroflavone), quercetin-3-O-(2″-acetyl)-glucosylgalactoside (flavonol), cyanidin-3-O-glucoside (kuromanin), and cyanidin-3-O-(6″-malonylglucoside) (two anthocyanins) showed a dramatically light-induced pattern, whereas two proanthocyanidins valoneoyl-glucose and procyanidin A6 showed an opposite pattern (Table 1).

Moreover, the total content of six types of flavonoids in different samples was analyzed based on the peak area of each compound. As shown in Figure 3C, flavonols were the most abundant, which also had the largest number; they slightly increased in S2 and S3 rose petals and continued to rise in S4 petals. There was no obvious difference between S2 and S2D, but compared to S1 petals, S2D petals had a little higher content of flavonols. Proanthocyanidins were the second abundant in content, which increased in S2 petals, then slightly descended in S3 flowers and remained unchanged in S4 but showed no change in S2D compared to S1. As the second-largest number of flavonoid metabolites was detected in rose petals, flavones content also saw a sustained increase when exposed to light; however, it declined under shading light in S2D. Furthermore, the content of anthocyanin ceased to increase under shading, but continued to rise with illumination. Flavaonols exhibited no significant changes during rose flower development under both treatments. Isoflavones were the lowest in quantity and content, and they showed a declining trend from S1 to S3, then a slight rise at S4; however, they were expressed higher in S2D than that in S2 petals, but lower than S1 petals (Figure 3C).

### 2.3. Differential Metabolite Screening, Functional Annotation, and Enrichment Analysis

**Figure 4:**
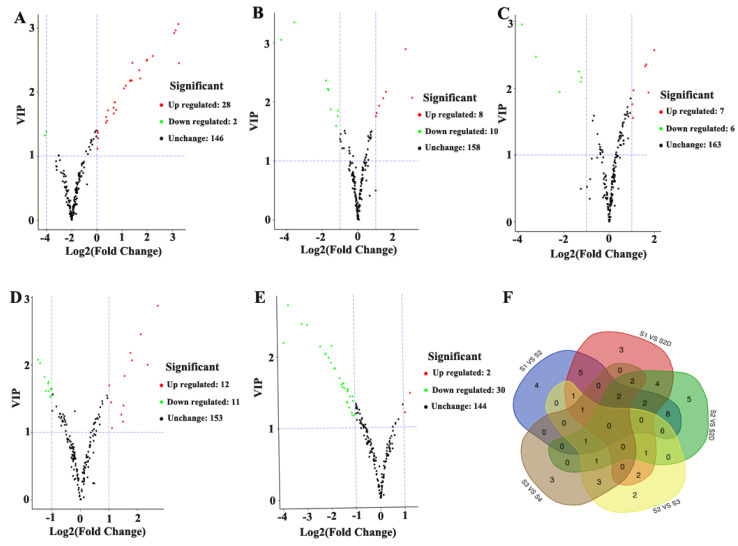
Differential flavonoid metabolites screening results for each comparison group by volcano plots ((**A**), S1 versus S2; (**B**), S2 versus S3; (**C**), S3 versus S4; (**D**), S1 versus S1D and (**E**), S2 versus S2D), and Venn diagram of these five comparison groups (**F**).

Differential flavonoid metabolites of each comparison group were screened by combing fold change (≥2 or ≤0.5) and variable importance in project (VIP, ≥1) of orthogonal signal correction and partial least squares-discriminant analysis (OPLS-DA). In total, 56 significantly differential metabolites (SDM) were screened from all the five comparison groups, including S1 VS S2, S2 VS S3, S3 VS S4, S1 VS S2D and S2 VS S2D, (Table 1). There were 30 SDM in S1 VS S2 group (28 up-regulated, 2 down-regulated), 18 between S2 and S3 (8 up-regulated, 10 down-regulated), 13 in S3 VS S4 group (7 up-regulated, 6 down-regulated), 23 between S1 and S2D (12 up-regulated, 11 down-regulated), and 32 between S2 and S2D (2 up-regulated, 30 down-regulated), (Figure 4A–E). Venn diagram showed the intersection of each comparison group. However, no common differential metabolites were observed among these five comparison groups with only two metabolites showing a continued change during rose development without covering, and each comparison group had its unique differential metabolites (Figure 4F).

**Figure 5:**
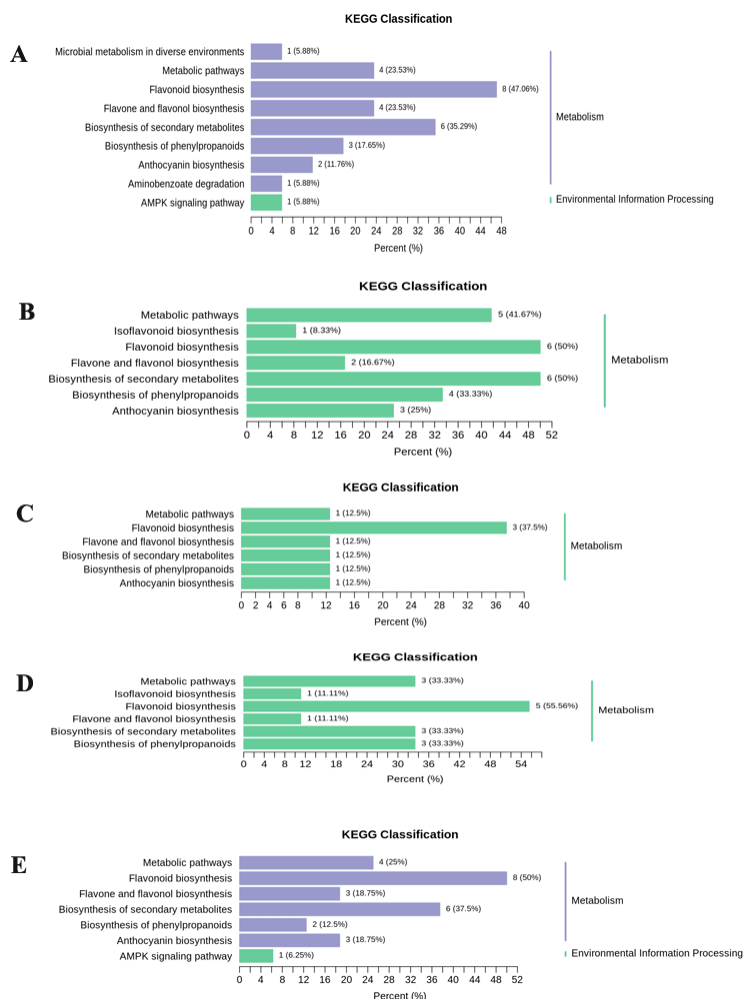
The differential flavonoid metabolite KEGG classification of each comparison group, (**A**) S1 versus S2; (**B**) S2 versus S3; (**C**) S3 versus S4; (**D**) S1 versus S1D; and (**E**) S2 versus S2D.

After annotation by the Kyoto Encyclopedia of Genes and Genomes (KEGG) database, the differential flavonoid metabolites of each comparison group were mainly enriched in pathways including isoflavones, flavonoid, flavone and flavonol, phenylpropanoids, secondary metabolites, anthocyanin, and metabolic pathways (Figure 5).

### 2.4. The Differential Metabolites in Developing Flowers Enriched in Flavonoid Pathway

**Figure 6:**
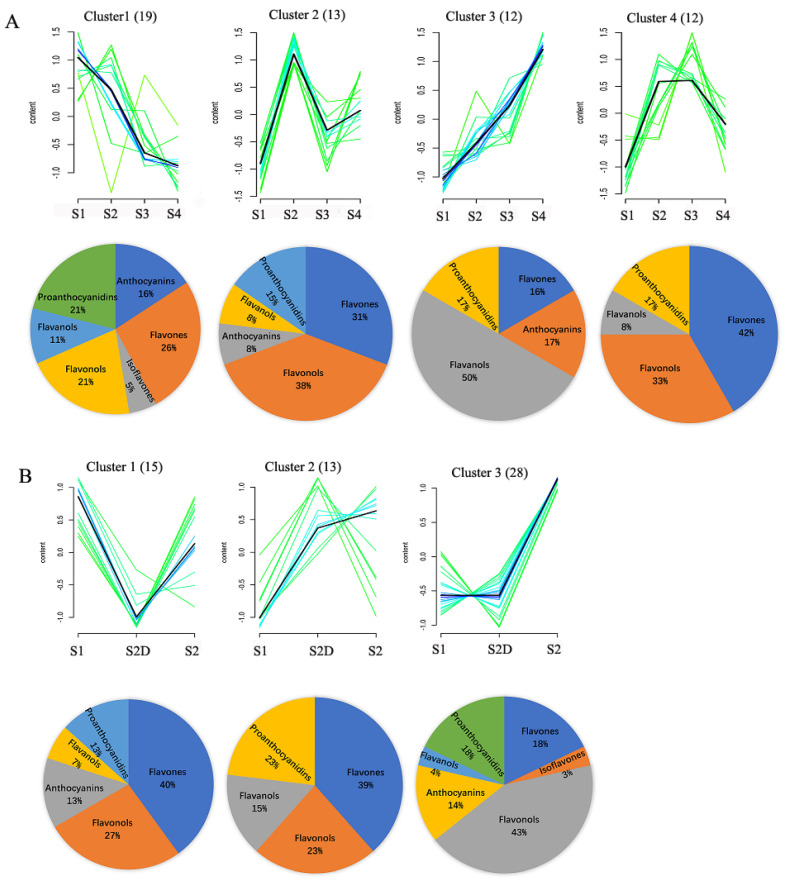
Sequence chart of differential metabolites in developing rose flowers and the ratio of metabolic groups for each cluster depicted above. (**A**) Rose flowers from S1 to S4 under natural light condition and (**B**) rose flowers from S1, S2D and S2.

The changing trends of these differential flavonoid metabolites (DFM) in developmental rose flowers were demonstrated by a sequence chart based on their relative content (Figure 6). For DFM in four developmental stages flowers of ‘Chen Xi’ under natural conditions, cluster 1 contained the greatest number of DFM (19), which had the highest content in S1 petals, but continued to decline with flower development. There were five flavones, four flavonols, two flavanols, one isoflavones, three anthocyanins, and four proanthocyanidins in this cluster (Figure 5A). In cluster 2, DFM, including five flavonols, four flavones, one flavanol, one anthocyanins, and two proanthocyanidins, had the lowest expression in S1 petals, then rapidly increased in S2 petals and decreased in S3 petals, a little up-regulated in S4 petals. However, most of the DFM in cluster 3 were up-regulated constantly along with rose flower development, and five flavones, four flavonols, one flavanol, and two proanthocyanidins belonged to this cluster. Unlike the other three clusters, DFM in cluster 4 had the lowest content in S1 petals, then sharply rose in S2 petals, and maitained in S3 petals, declined S4 petals (Figure 6A).

Moreover, petals under the shading treatment in stage 2 (S2D) had different changes compared with petals under the natural light condition in S2, which were classified into 3 clusters (Figure 5B). DFM in cluster 1 were down-regulated in S2D petals, but up-regulated in S2 petals. This cluster contained 15 members, and flavones took up the greatest percentage (40%), followed by flavonols (27%) (Figure 5B). In cluster 2, DFM both in S2 and S2D petals were up-regulated compared to S1 petals, but DFM had higher content in S2 petals than in S2D petals. Flavones were the most abundant in cluster 2 (39%), then followed by flavonols and proanthocyanidins (23%). In addition, cluster 3 contained the greatest number of DFM (28), which showed no significant change between S1 and S2D petals, but sharply accumulated in S2 petals, and six type of flavonoids were contained in this cluster; among them, flavonols were the most abundant (Figure 6B).

**Figure 7:**
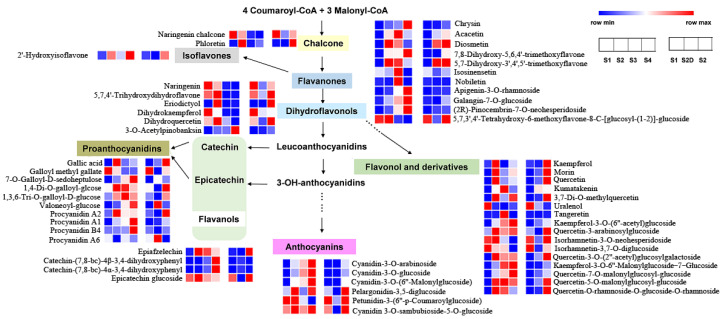
Differential metabolite enrichment in flavonoid biosynthesis and their accumulation profiles in developing rose flowers by heatmap.

All the DFM were enriched in the flavonoid biosynthesis pathway (Figure 7). There were two chalcones; one was the most abundant in S1 flowers, and decreased in the following development stages, and it was expressed more in S2 petals than in S2D petals. The other one increased sharply from S1 to S2 but declined quickly in flowers S3 and S4. Moreover, compared to the S1 flower, it exhibited no change in the S2 flower without illumination. Only one type of isoflavone showed differences among samples, and it showed a strange change trend, which had lower content in S1 flowers, then rose in S2 flowers, but decreased in S3 flowers and increased again and reached the highest content in S4 flowers, while it had lower content in S2D flowers. Among the 11 flavanones, the content of five members increased along with flower development, while they showed no change between S1 and S2D flowers. Only one had higher content in S1 flower, two of them showed higher content in S2D flower, and two had the highest content in S3 flowers. Six dihydroflavonols showed a different expression trend; five of them were more abundant in S1 and S2 flowers, and the remaining one was only expressed highly in S4 flowers and had lower content in other stages’ flowers. The number of flavonol and derivatives is the largest (16); three of them had a higher content in S1 flowers and most members had a higher content in the flowers of S2, S3, and S4. However, all the members showed lower expression levels in S2D. Four flavanols displayed differential expression levels among different development of flowers, two of them had lower content in the first three stages, but quickly increased in S4 flowers; one was expressed the lowest only in S2D, and one had a lower content in S1 and S2D flowers, then ascended quickly in S2 flowers and slowly decreased in S3 and S4 flowers. The last one was expressed higher in S1 and S2 flower, and a slight reduction in S3 and S4 flowers. Ten proanthocyanidins were detected with differentially expressed levels among samples, and most of them had higher content in the flowers of S2, S3, and S4, several of them expressed more in S1 flowers or S2 flowers. There were six anthocyanins that showed different expression patterns during rose flower development. Three of them displayed a gradual increase along with flower development, while the other three had higher expression levels in S2 flowers. However, all of them had lower expression in S2 flowers without illumination (Figure 7).

Overall, during the petal color changing progress, most of the flavonoid metabolites were accumulated from S1 to S2; only two flavonols exhibited a significant decrease. Eight compounds including three flavones, two flavonols, one flavanol, and two anthocyanins were decreased from S2 to S3, while eight flavones and one anthocyanin significantly increased from S2 to S3. From S3 to S4, seven compounds (one flavone, two flavonols, two flavanols, one anthocyanin, and one proanthocyanidin) and six compounds (three flavonols, two flavones, and one anthocyanin) were significantly increased and decreased, respectively (Table 1).

When treated by shading, 11 flavonoid compounds (5 flavonols, 4 flavones and 2 proanthocyaidins) significantly increased from S1 to S2D petals, but almost all of them decreased in the S2D vs. S2 comparison group (Table 1). Except for two proanthocyanidns (valoneoyl-glucose and procyanidin A6), whose content was obviously more abundant in S2D petals compared with that in S2 and S1 and S2, while the other 30 compounds (5 flavones, 1 isoflavone, 13 flavonols, 2 flavanols, 6 anthocyanins and 3 proanthocyanidins) were decreased greater in S2D petals than in S2 petals.

Since the red color was determined by anthocyanins, based on the accumulation profiles of these six anthocyanins, although all of their content sharply declined in shading petals compared with S2 petals, only three cyanidin derivatives (anidin-3-*O*-arabinoside, cyanidin-3-*O*-glucoside and cyanidin-3-*O*-(6″-Malonylglucoside)) exhibited sustained rapid accumulation during flower blooming, and keep nearly the same content in S1 and S2D (Table 1, Figure 7), which was consistent with the color. Thus, we speculated that these three anthocyanins mainly contributed to the red color of petals.

## 3. Discussion

The ‘Chen Xi’ rose, whose petal colors can be changed during the flowering process, has highly ornamental value. Previous studies reported that the yellow color of the rose is determined by carotenoids, while the red color is mainly caused by anthocyanins [3,10]. Furthermore, rose petals have been widely used as traditional medicinal therapy, food additives, as well as for cosmetics due to these abundant bioactive compounds [4,11,16]. Here, we detected the flavonoid metabolites during different developmental stages of this bicolored rose flower, with one stage (S2) subjected to shading treatment.

### 3.1. Abundant Flavonoid Metabolites Accumulated in Rose Flower Petals

In total, 176 flavonoid metabolites were identified, containing 49 flavones, 59 flavonols, 12 flavanones, 3 isoflavones, 12 anthocyanins, and 41 proanthocyanidins in this bicolored rose flower (Table S1). Among them, many metabolites were found for the first time. Previous studies identified four anthocyanin compounds, including cyanidin 3-*O*-glucoside, pelargonidin 3-glucoside, cyanidin 3,5-di-*O*-glucoside and pelargonidin 3,5-di-O-glucoside [10,11,12], which belongs to single or di-glycoside derivatives of cyanidin and pelargonidin. However, in this study, only two anthocyanins were the same as before (cyanidin-3-*O*-glucoside and pelargonidin-3,5-diglucoside), and five other cyanidins and one petunidin were glycosylated by other types of glycosides, including arabinoside, malonylglucoside, sambubioside, and coumaroylglucoside with single or diosaccharide. Other kinds of anthocyanin compounds with different glucosides were detected, such as delphinidin-3-*O*-glucoside, malvidin-3-*O*-malonylglucoside and petunidin-3-(6″-p-Coumaroylglucoside) (Table S1). Only 20 flavones, including 16 kaempferol and derivatives and 3 quercetins and derivatives as well as one flavan-3-ol derivative, were identified before [10]; however, 59 flavonols and other flavonoid compounds were detected (Table S1) which border the flavonoid metabolites in rose flowers. These results showed that rose flowers are rich in flavonoid metabolites, with their content being high during the blooming stages. Flavonoids, especially the high content of flavonols in rose flowers, were reported to have several bioactivities, such as antioxidant, anti-inflammatory, and anticancer roles [17,18,19]. Moreover, yellow rose flowers contain abundant carotenoids [10], which play vital roles in human and animal nutrition and in reducing the risk of vitamin A deficiency, cancer, and cardiovascular diseases [20,21]. Therefore, ‘Chen Xi’ flowers may have a higher medicine value for being rich in both flavonoids and carotenoids.

### 3.2. Light Affects Certain Flavonoid, Especially Anthocyanin, Biosynthesis of Rose Flowers

Light is important for plant growth and photomorphogenesis, and it affects the accumulation of pigment in plant organs, including fruit skin, such as pear [13,22], grape [15], strawberry [23], and apple [14] and flower petals, such as Lily [24], Lisianthus [25], Gerbera [26]. These organs’ color is due to anthocyanin pigment whose biosynthesis is dependent on light induction.

In this study, the rose flower bud was yellow, but the part which was exposed to sunlight turned red, and the petals become redder along with the blooming stage after it totally opens, shadowing the yellow color. However, when the flower was grown under the shade, the flower remained yellow, which was consistent with anthocyanin accumulation. Therefore, it can be inferred that light promotes anthocyanin accumulation in rose petals, but cannot affect carotenoids biosynthesis. Furthermore, the light also affects other flavonoid compounds’ biosynthesis, since most of them increased with the rose flower development under sunlight. It is a fantastic phenomenon, and we will identify this hypothesis from gene expression level in the next step, and study how light induces anthocyanin biosynthesis. It will be an interesting subject worth exploring further.

## 4. Materials and Methods

### 4.1. Plant Materials and Sample Collection

Roses were planted in the rose display area of Hubei University (30°56′ N, 114°32′ E). The flower development from little bud to color fading was detected in April 2020 (Figure 1A). After the flower was fully opened, two different light treatments were carried out: natural sunlight (control group) and shading (Figure 1B). The shading treatment was carried out by wrapping the flower with a black opaque paper bag. Rose flower petals at four development stages (S1–S4), which were defined in Section 2.1, and one stage of flowers (corresponding to S2 flower) under light shading (Figure 1B and Figure S1(S2D)) were collected as samples. Three biological replicates were performed for each sample. The samples were then frozen in liquid nitrogen immediately and stored at −80 °C for further metabolite analysis.

### 4.2. Sample Preparation and Extraction

The freeze-dried rose petals were crushed by a mixer mill (MM 400, Retsch, Laichi, Germany) with a zirconia bead at 30 Hz for 1.5 min. About 100 mg of powder was weighted and extracted at 4 °C with 1.0 mL 70% aqueous methanol overnight. After being centrifuged at 10,000 g for 10 min, the extracts were absorbed (CNWBOND Carbon-GCBSPE Cartridge, 250 mg, 3 mL; ANPEL, Shanghai, China, www.anpel.com.cn/cnw, accessed on 23 September 2020) and filtrated using 0.22 μm filter (SCAA-104, ANPEL, Shanghai, China, http://www.anpel.com.cn/, accessed on 23 September 2020) LC-MS analysis.

### 4.3. HPLC Conditions

HPLC analysis was carried according to the method described by Wang et al. [27]. A 5 μL volume of each sample extract was analyzed using an LC-ESI-MS/MS system (HPLC, Shim-pack UFLC SHIMADZU CBM30A system, www.shimadzu.com.cn/, accessed on 23 September 2020; MS, Applied Biosystems 4500 Q TRAP, www.appliedbiosystems.com.cn/, accessed on 23 September 2020). The analytical conditions were set as follows, chromatographic column, Waters ACQUITY UPLC HSS T3 C18 (1.8 μm, 2.1 mm × 100 mm); solvent system, water (containing 0.04% acetic acid): acetonitrile (containing 0.04% acetic acid); the gradient program, 100:0 V/V at 0 min, 5:95 V/V at 11.0 min, 5:95 V/V at 12.0 min, 95:5 V/V at 12.1min, 95:5 V/V at 15.0 min; flow rate, 0.40 mL/min; temperature, 40 °C. The effluent was alternatively connected to an ESI-triple quadrupole-linear ion trap (QTRAP)-MS.

### 4.4. ESI-Q TRAP-MS/MS

Mass spectrometry was conducted following the protocol described previously [28,29]. The API 4500 triple quadrupole-linear ion trap mass spectrometer (Q TRAP) LC/MS/MS System, coupled with an ESI Turbo Ion-Spray interface was applied to generate the LIT and triple quadrupole (QQQ) scans, which is operating in a positive ion mode under the control of Analyst 1.6.3 software (AB Sciex). The operation parameters of the ESI source were set as below: ion source, turbo spray; source temperature, 550 °C; ion spray voltage (IS), 5500 V; ion source gas I (GSI), gas II (GSII), curtain gas (CUR) was set at 55, 60, and 25.0 psi, respectively; the collision gas (CAD) was high. Instrument tuning and mass calibration were performed with 10 and 100 μmol/L polypropylene glycol solutions in QQQ and LIT mode, respectively. QQQ scans were acquired as MRM experiments with collision gas (nitrogen) set to 5 psi. Decluttering potential (DP) and collision energy (CE) for individual MRM transitions were performed with further DP and CE optimization. Based on the metabolites eluted within each period, we monitored a specific set of MRM transitions.

### 4.5. Qualitative and Quantitative Analysis of Metabolites from Rose Flowers

A widely targeted metabolome method constructed by Metware Biotechnology Co., Ltd. (Wuhan, China) (http://www.metware.cn/, accessed on 23 September 2020) was applied to qualitatively analyze the flavonoid metabolites based on both the public available and self-built database MWDB. Additionally, a multiple reaction monitoring (MRM) method was applied for the quantification analysis of metabolites, which, using the peak area of each chromatographic peak, represents the relative content of the corresponding substance, and the information of metabolite retention time and peak pattern was used to calibrate the peaks of mass spectra detected for each compound in different samples.

### 4.6. Statistical Analysis

Three biological replicates were performed in the present study. Hierarchical clustering analysis and principal component analysis (PCA) were carried out by R software (http://www.r-project.org/, accessed on 23 September 2020). The analysis on the content of different kinds of flavonoid was performed using Microsoft Office Excel 2016 (Microsoft Corporation., Redmond, WA, USA).

## 5. Conclusions

Taken together, our study revealed that light induces pigmentation changes in the petals of the ‘Chen Xi’ rose variety. When exposed to light, the ‘Chen Xi’ rose flower color changed from yellow to red, but the flower remains yellow under darkness. Flavonoid metabolites were detected during the ‘Chen Xi’ flower color changing process, among a total of 176 flavonoid compounds, 56 compounds showed different accumulation profiles, including 19 flavonols, 16 flavones, 1 isoflavone, 4 flavanols, 6 anthocyanins, and 10 proanthocyanidins. Some compounds increased, while some decreased accumulation during flower development; however, the total content of most of them were increased, especially flavonols, flavones and anthocyanins. Anthocyanins were the main compounds, especially three cyanidin derivatives that determined the flower’s red color. These results lay a foundation for further study on the mechanism of light-induced anthocyanin biosynthesis and facilitate to breed new rose cultivar colors.

## Figures and Tables

**Table 1:** The information of differential metabolites from five comparison groups.

Index	MW (Da)	Ionization Model	Compounds	Class	S2/S1	S3/S2	S4/S3	S2D/S1	S2D/S2
pme2960	272.07	[M^+^H]^+^	Naringenin chalcone *	Chalcones	0.72 /	0.29	0.70 /	0.38	0.52 /
pme1201	274.08	[M^−^H]^−^	Phloretin	Chalcones	3.29	0.31	1.44 /	0.93 /	0.28
pme0376	272.07	[M^−^H]^−^	Naringenin	Dihydroflavone	0.69 /	0.29	0.78 /	0.36	0.52 /
Hmqp004476	272.07	[M^+^H]^+^	5,7,4′-Trihydroxydihydroflavone *	Dihydroflavone	1.15 /	0.32	1.06 /	0.66 /	0.57 /
mws0064	288.06	[M^−^H]^−^	Eriodictyol	Dihydroflavone	19.10	0.05	1.75 /	1.29 /	0.07
mws1094	288.06	[M^−^H ^−^	Dihydrokaempferol	Dihydroflavonol	0.50	0.09	0.42	0.46	0.92 /
mws0044	304.06	[M^−^H]^−^	Dihydroquercetin (Taxifolin)	Dihydroflavonol	1.31 /	0.60 /	0.63 /	0.61 /	0.47
mws1174	314.08	[M^−^H]^−^	3-*O*-Acetylpinobanksin	Dihydroflavonol	7.90	1.06 /	3.98	2.84	0.36
mws0040	254.06	[M^+^H]^+^	Chrysin	Flavones	6.42	1.35 /	3.33	2.11	0.33
mws0051	284.07	[M^+^H]^+^	Acacetin	Flavones	2.57	1.49 /	0.55 /	2.15	0.84 /
mws0058	300.06	[M^−^H]^−^	Diosmetin	Flavones	3.16	0.83 /	0.51 /	2.82	0.89 /
Zmhp004065	344.09	[M^+^H]^+^	7,8-Dihydroxy-5,6,4′-trimethoxyflavone *	Flavones	6.58	0.99 /	1.79 /	0.87 /	0.13
mws1474	344.09	[M^+^H]^+^	5,7-Dihydroxy-3′,4′,5′-trimethoxyflavone	Flavones	5.22	0.93 /	0.56 /	5.11	0.98 /
pmp001076	372.12	[M^+^H]^+^	Isosinensetin *	Flavones	0.88 /	1.99 /	0.22	0.62 /	0.70 /
mws0043	402.13	[M^+^H]^+^	Nobiletin	Flavones	0.83 /	6.91	0.11	0.77 /	0.93 /
pmn001668	416.15	[M^−^H]^−^	Apigenin-3-*O*-rhamnoside	Flavones	1.56 /	2.28	1.74 /	1.40 /	0.90 /
Lmlp005572	432.11	[M^+^H]^+^	Galangin-7-*O*-glucoside *	Flavones	2.72	1.64 /	1.45 /	0.61 /	0.22
HJN076	564.18	[M^−^H]^−^	(2R)-Pinocembrin-7-*O*-neohesperidoside	Flavones	5.08	2.97	1.76 /	4.32	0.85 /
Lmnp002448	640.16	[M^+^H]^+^	5,7,3′,4′-Tetrahydroxy-6-methoxyflavone-8-C-[glucosyl-(1-2)]-glucoside	Flavones	1.05 /	0.35	0.77 /	0.42	0.40
Lmgn002843	286.05	[M^−^H]^−^	2′-Hydroxyisoflavone	Isoflavones	1.97 /	0.73 /	1.63 /	0.80 /	0.41
mws1068	286.05	[M^−^H]^−^	Kaempferol *	Flavonols	2.03	0.43	1.55 /	0.94 /	0.46
pme3514	302.04	[M^−^H]^−^	Morin *	Flavonols	3.40	0.50 /	1.39 /	1.45 /	0.43
pme2954	302.04	[M^+^H]^+^	Quercetin	Flavonols	3.18	0.52 /	1.20 /	1.45 /	0.46
mws0038	314.08	[M^−^H]^−^	Kumatakenin	Flavonols	2.57	1.58 /	0.42 /	2.69	1.05 /
mws0917	330.07	[M^−^H]^−^	3,7-Di-*O*-methylquercetin	Flavonols	5.31	0.46	2.02	1.40 /	0.26
pmp000365	370.11	[M^+^H]^+^	Uralenol	Flavonols	0.48	0.91 /	1.19 /	0.63 /	1.32 /
mws0055	372.12	[M^+^H]^+^	Tangeretin	Flavonols	1.15 /	8.18	0.07	0.83 /	0.72 /
Lmmn003398	490.11	[M^−^H]^−^	Kaempferol-3-*O*-(6″-acetyl)glucoside	Flavonols	2.03	1.35 /	1.21 /	1.56 /	0.77 /
Lmln001951	596.14	[M^−^H]^−^	Quercetin-3-arabinosylglucoside *	Flavonols	1.61 /	0.84 /	1.10 /	0.70 /	0.44
pme1540	624.17	[M^+^H]^+^	Isorhamnetin-3-*O*-neohesperidoside	Flavonols	1.08 /	0.55 /	0.43	0.43	0.40
Hmcp001578	640.16	[M^+^H]^+^	Isorhamnetin-3,7-*O*-diglucoside *	Flavonols	0.78 /	0.99 /	0.59 /	0.50	0.64 /
Hmln001682	668.16	[M^−^H]^−^	Quercetin-3-*O*-(2″-acetyl)-glucosylgalactoside	Flavonols	18.83	0.73 /	1.04 /	6.54	0.35
Hmcp001629	696.15	[M^+^H]^+^	Kaempferol-3-*O*-(6″-Malonylglucoside)-7-*O*-Glucoside	Flavonols	7.97	2.67	1.12 /	1.93 /	0.24
pmb0709	712.15	[M^+^H]^+^	Quercetin-7-*O*-malonylglucosyl-glucoside *	Flavonols	4.98	1.39 /	1.56 /	2.92	0.59 /
pmb0706	712.15	[M^+^H]^+^	Quercetin-5-*O*-malonylglucosyl-glucoside	Flavonols	9.37	1.05 /	0.83 /	3.50	0.37
Hmcp001757	756.21	[M+H]+	Quercetin-*O*-rhamnoside-O-glucoside-O-rhamnoside	Flavonols	3.26	0.89 /	0.61 /	1.19 /	0.36
mws1422	274.08	[M^+^H]^+^	Epiafzelechin *	Flavanols	4.22	0.89 /	0.74 /	0.81 /	0.19
pmn001415	452.11	[M^−^H]^−^	Catechin-(7,8-bc)-4β-(3,4-dihydroxyphenyl)-dihydro-2-(3H)-ne *	Flavanols	0.94 /	2.01	3.10	1.44 /	1.53 /
pmn001416	452.11	[M^−^H]^−^	Catechin-(7,8-bc)-4α-(3,4-dihydroxyphe-nyl)-dihydro-2-(3H)-ne	Flavanols	1.09 /	1.70 /	3.02	1.47 /	1.35 /
HJN041	452.13	[M^−^H]^−^	Epicatechin glucoside	Flavanols	1.12 /	0.81 /	0.94 /	0.46	0.41
Smlp002532	419.10	[M]^+^	Cyanidin-3-*O*-arabinoside	Anthocyanins	4.44	1.33 /	1.52 /	0.97 /	0.22
pmb0550	449.11	[M]^+^	Cyanidin-3-*O*-glucoside (Kuromanin)	Anthocyanins	17.48	2.07	1.39 /	1.36 /	0.08
pmb0542	535.11	[M]^+^	Cyanidin-3-*O*-(6″-Malonylglucoside)	Anthocyanins	16.81	6.33	1.78 /	1.91 /	0.11
pme1793	595.17	[M]^+^	Pelargonidin-3,5-diglucoside	Anthocyanins	1.76 /	0.46	2.08	0.51 /	0.29
Lmpp003815	625.16	[M]^+^	Petunidin-3-(6″-p-Coumaroylglucoside)	Anthocyanins	0.98 /	0.58 /	0.40	0.48	0.49
Lmjp001323	743.20	[M]^+^	Cyanidin 3-*O*-sambubioside-5-*O*-glucoside	Anthocyanins	1.19 /	0.83 /	1.19 /	0.49	0.41
mws0024	170.02	[M^−^H]^−^	Gallic acid	Proanthocyanidins	2.66	0.51 /	1.17 /	1.40 /	0.53 /
pmn001519	336.05	[M^−^H]^−^	Galloyl Methyl gallate	Proanthocyanidins	0.78 /	0.75 /	1.01 /	0.50	0.64 /
Cmsn000894	362.08	[M^−^H]^−^	7-O-Galloyl-D-sedoheptulose	Proanthocyanidins	1.52 /	1.38 /	1.41 /	2.01	1.32 /
Wmhn001495	484.08	[M^−^H] ^−^	1,4-Di-O-galloyl-glcose	Proanthocyanidins	1.34 /	0.97 /	0.82 /	0.64 /	0.47
Lmfn001209	636.10	[M^−^H]^−^	1,3,6-Tri-O-galloyl-D-glucose *	Proanthocyanidins	1.82 /	1.30 /	0.76 /	0.47	0.26
Cmhn000855	650.08	[M^−^H]^−^	Valoneoyl-glucose	Proanthocyanidins	1.39 /	1.63 /	1.39 /	3.36	2.42
pme0432	576.13	[M^−^H]^−^	Procyanidin A2 *	Proanthocyanidins	2.07	0.70 /	1.02 /	0.73 /	0.35
pme0430	576.13	[M^−^H]^−^	Procyanidin A1	Proanthocyanidins	2.06	1.13 /	1.56 /	1.21 /	0.59 /
pmn001667	578.14	[M^−^H]^−^	Procyanidin B4 *	Proanthocyanidins	1.03 /	1.07 /	2.11	0.60 /	0.58 /
HJN074	592.16	[M^−^H]^−^	Procyanidin A6	Proanthocyanidins	0.66 /	1.50 /	0.86 /	1.37	2.08

* Indicate there are several different isomerides of the marked compounds. / means not changed.

## Data Availability

The data presented in this study are available in supplementary material.

## Supplementary Materials

The following is available online at https://www.mdpi.com/article/10.3390/plants10102065/s1, Table S1: The information of the flavonoid metabolites identified in rose petals. Figure S1: The HPLC chromatograms of flavonoid metabolites from rose petal samples.
