# Supplementary material for: Metabolomic Analysis on the Petal of ‘Chen Xi’ Rose with Light-Induced Color Changes
Source: Plants (Basel). 2021 Sep 30;10(10):2065. doi: 10.3390/plants10102065 (PMC8541279; doi:10.3390/plants10102065)

Table S1 The information of the flavonolids metabolites identified in rose petals.

Class	Index	The[a] MW[b] (Da)	Exp[c] MW[b] (Da)	Ion model	MS[2] (Da)	Compounds	S1	S2	S3	S4	DS2	Group
**Flavones**	mws0040▲	254.06	255.07	[M+H]+	153.00	Chrysin	3.91E+03	2.51E+04	3.40E+04	1.13E+05	8.25E+03	I
	mws0789▲	256.07	255.07	[M-H]-	151.00	Pinocembrin(Dihydrochrysin)	2.05E+04	3.55E+04	2.92E+04	5.54E+04	2.58E+04	I
	pme0376▲	272.07	271.06	[M-H]-	151.00	Naringenin	9.55E+04	6.63E+04	1.94E+04	1.51E+04	3.43E+04	V
	pme2960▲	272.07	273.08	[M+H]+	153.00	Naringenin chalcone*	2.60E+05	1.87E+05	5.47E+04	3.81E+04	9.79E+04	IV
	Hmqp004476▲	272.07	273.08	[M+H]+	153.02	5,7,4'-Trihydroxydihydroflavone*	1.23E+05	1.42E+05	4.58E+04	4.87E+04	8.11E+04	V
	pme1201▲	274.08	273.08	[M-H]-	167.00	Phloretin	8.52E+03	2.81E+04	8.76E+03	1.26E+04	7.96E+03	II
	mws0051▲	284.07	285.08	[M+H]+	270.00	Acacetin	1.47E+03	3.78E+03	5.61E+03	3.10E+03	3.17E+03	II
	pme0088▲	286.05	285.04	[M-H]-	151.00	Luteolin	5.45E+03	6.61E+03	7.84E+03	1.18E+04	4.66E+03	I
	mws0064▲	288.06	287.06	[M-H]-	135.00	Eriodictyol	3.09E+03	5.90E+04	3.01E+03	5.27E+03	3.98E+03	II
	mws0058▲	300.06	299.06	[M-H]-	284.00	Diosmetin	1.68E+04	5.31E+04	4.41E+04	2.25E+04	4.74E+04	II
	mws0920▲	302.04	301.04	[M-H]-	151.00	Tricetin	3.42E+04	3.44E+04	3.31E+04	2.91E+04	3.35E+04	V
	pmp000106▲	312.10	313.11	[M+H]+	298.10	5,7,4'-Trimethoxyflavone	2.09E+03	1.76E+03	2.60E+03	1.80E+03	1.85E+03	III
	Zmhp004065▲	344.09	345.10	[M+H]+	330.07	7,8-Dihydroxy-5,6,4'-trimethoxyflavone*	1.39E+03	9.17E+03	9.06E+03	1.62E+04	1.22E+03	I
	mws1474▲	344.09	345.10	[M+H]+	284.00	5,7-Dihydroxy-3',4',5'-trimethoxyflavone	2.58E+03	1.34E+04	1.24E+04	6.98E+03	1.32E+04	I
	pmp001076▲	372.12	372.12	[M+H]+	343.08	Isosinensetin*	1.71E+03	1.51E+03	3.01E+03	6.62E+02	1.06E+03	III
	Lmsp010424▲	372.12	373.13	[M+H]+	211.02	3,4',5,6,7-Pentamethoxyflavone*	2.38E+03	2.74E+03	3.15E+03	3.25E+03	1.62E+03	I
	mws0043▲	402.13	373.13	[M+H]+	373.10	Nobiletin	3.82E+04	3.18E+04	2.20E+05	2.35E+04	2.95E+04	III
	pmn001668▲	416.15	415.14	[M-H]-	161.00	Apigenin-3-O-rhamnoside	8.27E+03	1.29E+04	2.95E+04	5.12E+04	1.16E+04	I
	Lmyp004617▲	418.13	419.13	[M+H]+	257.08	Pinocembrin 7-O-glucoside(Pinocembroside)	9.32E+06	1.23E+07	1.32E+07	1.27E+07	8.63E+06	II
	Lmlp005572▲	432.11	433.11	[M+H]+	271.06	Galangin-7-O-glucoside*	2.53E+05	6.88E+05	1.13E+06	1.63E+06	1.54E+05	I
	mws1179▲	434.12	433.11	[M-H]-	271.00	Naringenin-7-O-glucoside	1.55E+06	1.56E+06	1.53E+06	1.91E+06	1.32E+06	IV
	mws2118▲	436.14	435.13	[M-H]-	166.70	Phlorizin	2.84E+06	2.70E+06	2.71E+06	2.83E+06	2.28E+06	V
	Lmlp003531▲	448.10	449.11	[M+H]+	287.06	Luteolin-3'-O-glucoside*	8.60E+05	8.53E+05	8.38E+05	9.07E+05	8.27E+05	II

HJN086▲	450.12	449.11	[M-H]-	287.06	Eriodictyol-3'-O-glucoside	8.60E+05	8.16E+05	7.23E+05	7.33E+05	5.90E+05	IV
mws4167▲	462.08	463.09	[M+H]+	287.06	Luteolin-7-O-glucuronide	2.44E+06	2.74E+06	3.10E+06	2.91E+06	1.67E+06	I
Zmhn003191▲	462.12	461.11	[M-H]-	341.06	Isoscoparin	2.17E+04	1.85E+04	2.01E+04	1.72E+04	1.98E+04	III
pmb3012▲	462.12	461.11	[M-H]-	299.20	Chrysoeriol-7-O-glucoside	5.01E+04	5.38E+04	4.69E+04	4.96E+04	4.46E+04	II
pmp000579▲	462.12	463.12	[M+H]+	301.07	Diosmetin-7-O-galactoside	6.05E+04	1.03E+05	7.77E+04	8.12E+04	6.58E+04	II
Xmsn002700▲	466.11	465.13	[M-H]-	303.06	Taxifolin-3'-O-glucoside	9.84E+05	1.10E+06	9.24E+05	1.01E+06	1.14E+06	VI
pmb3000▲	504.13	503.12	[M-H]-	341.10	Chrysoeriol-O-acetylglucoside	2.74E+04	3.16E+04	3.46E+04	2.79E+04	2.61E+04	I
Zmsp004363▲	506.11	507.12	[M+H]+	303.05	Quercetin-acetyl-glucoside*	7.39E+05	8.94E+05	9.49E+05	1.38E+06	6.15E+05	I
pmb3041▲	522.10	521.09	[M-H]-	329.20	Tricin-O-saccharic acid	3.16E+04	4.01E+04	4.05E+04	4.55E+04	2.30E+04	II
pmp000587▲	534.10	535.11	[M+H]+	449.10	Luteolin-7-O-(6"-malonyl)glucoside	7.20E+03	1.20E+04	1.99E+04	2.48E+04	1.15E+04	I
HJN076▲	564.18	563.18	[M-H]-	255.07	(2R)-Pinocembrin-7-O-neohesperidoside	7.26E+03	3.69E+04	1.09E+05	1.92E+05	3.14E+04	I
pme3227▲	578.16	579.17	[M+H]+	433.00	Vitexin-2"-O-rhamnoside	4.94E+04	4.03E+04	3.41E+04	4.03E+04	4.32E+04	III
Lmyp004407▲	594.14	595.14	[M+H]+	287.05	Kaempferol-3-O-(2"-trans-p-Coumaroyl) galactoside*	1.00E+07	1.25E+07	1.21E+07	1.83E+07	6.47E+06	I
Hmjn004446▲	594.14	593.13	[M-H]-	285.04	Luteolin-7-O-(6"-caffeoyl)rhamnoside*	4.18E+06	4.34E+06	5.29E+06	6.20E+06	3.20E+06	II
Lmyp004318▲	594.14	595.14	[M+H]+	287.06	Kaempferol-3-O-(6"-trans-p-Coumaroyl) galactoside*	1.06E+07	1.33E+07	1.26E+07	1.90E+07	6.83E+06	I
pmp001079▲	594.16	595.17	[M+H]+	449.10	Luteolin-7-O-neohesperidoside (Lonicerin)	1.49E+05	1.55E+05	1.79E+05	2.32E+05	1.88E+05	VI
Lmsn002815	594.16	593.15	[M-H]-	285.04	Kaempferol-3-O-rutinoside (Nicotiflorin)	2.39E+05	2.25E+05	2.60E+05	3.55E+05	2.85E+05	III
Lmyp003500▲	600.11	601.12	[M+H]+	287.06	Kaempferol-3-O-(2"-galloyl)galactoside	1.48E+07	1.20E+07	1.47E+07	1.49E+07	1.43E+07	III
Lmyp003599▲	600.11	601.12	[M+H]+	287.06	Kaempferol-3-O-(6"-galloyl)glucoside*	7.78E+06	6.61E+06	5.85E+06	5.27E+06	8.20E+06	III
Lmyp004052▲	610.13	611.14	[M+H]+	303.05	Quercetin-3-O-(6"-trans-p-Coumaroyl)-galactoside	3.24E+06	3.92E+06	3.47E+06	5.85E+06	2.13E+06	I
pme0001▲	610.19	609.18	[M-H]-	301.00	Hesperetin-7-O-neohesperidoside (Neohesperidin)	9.39E+06	1.29E+07	1.32E+07	1.49E+07	1.03E+07	I

	Lmnp002448▲	640.16	641.17	[M+H]+	479.11	5,7,3',4'-Tetrahydroxy-6-methoxyflavone-8-C-[glucosyl-(1-2)]-glucoside	7.62E+04	8.00E+04	2.79E+04	2.14E+04	3.20E+04	V
	pmb0578▲	654.16	655.17	[M+H]+	207.00	Luteolin-7-O-(6"-sinapoyl)glucoside	1.25E+05	1.45E+05	1.57E+05	1.63E+05	1.36E+05	VI
	Hmmp002447▲	724.19	725.20	[M+H]+	433.11	Apigenin-O-rutinoside-O-glucoside	5.20E+04	4.48E+04	4.45E+04	3.78E+04	4.90E+04	III
	Lmyp003857▲	752.12	753.13	[M+H]+	287.06	Kaempferol-3-O-(2",6"-digalloyl)-glucoside	4.13E+05	2.91E+05	2.08E+05	1.58E+05	5.00E+05	VI
	Lmyp003588▲	768.12	769.12	[M+H]+	303.05	Quercetin-3-O-(2",6"-digalloyl)-glucoside	4.02E+05	3.72E+05	2.65E+05	2.21E+05	4.84E+05	IV
**Flavonols**	mws1068	286.05	285.04	[M-H]-	151.00	Kaempferol*	5.05E+04	1.02E+05	4.42E+04	6.83E+04	4.73E+04	II
	mws1094▲	288.06	287.06	[M-H]-	259.00	Dihydrokaempferol	4.19E+06	2.09E+06	1.79E+05	7.55E+04	1.92E+06	IV
	pme3514▲	302.04	301.04	[M-H]-	151.00	Morin*	8.56E+05	2.91E+06	1.46E+06	2.03E+06	1.24E+06	II
	pme2954	302.04	303.05	[M+H]+	137.02	Quercetin	8.16E+04	2.60E+05	1.34E+05	1.60E+05	1.18E+05	II
	mws0044▲	304.06	303.05	[M-H]-	125.02	Dihydroquercetin (Taxifolin)	6.49E+05	8.51E+05	5.09E+05	3.20E+05	3.99E+05	II
	mws1174▲	314.08	313.07	[M-H]-	253.00	3-O-Acetylpinobanksin	1.17E+04	9.25E+04	9.84E+04	3.91E+05	3.33E+04	I
	mws0038▲	314.08	313.07	[M-H]-	283.00	Kumatakenin	6.26E+03	1.61E+04	2.54E+04	1.07E+04	1.69E+04	I
	Lmdp003808▲	316.06	317.07	[M+H]+	302.04	Azaleatin (5-O-Methylquercetin)	1.36E+05	1.52E+05	1.39E+05	1.48E+05	1.89E+05	VI
	mws0917▲	330.07	329.07	[M-H]-	314.00	3,7-Di-O-methylquercetin	1.18E+03	6.29E+03	2.87E+03	5.81E+03	1.66E+03	I
	pmp000355▲	354.11	355.12	[M+H]+	299.05	Licoflavonol	1.48E+05	9.69E+04	9.87E+04	8.61E+04	1.29E+05	III
	pmp000365▲	370.11	371.11	[M+H]+	315.08	Uralenol	4.47E+04	2.14E+04	1.93E+04	2.29E+04	2.82E+04	IV
	mws0055▲	372.12	373.13	[M+H]+	343.00	Tangeretin	2.23E+04	2.56E+04	2.10E+05	1.46E+04	1.85E+04	III
	pmn001637	418.09	417.08	[M-H]-	285.00	Kaempferol-3-O-arabinoside (Juglanin)	5.17E+05	6.23E+05	6.65E+05	7.19E+05	4.74E+05	I
	pme0321	432.11	431.10	[M-H]-	285.00	Kaempferol-7-O-rhamnoside*	2.23E+06	2.36E+06	2.19E+06	2.38E+06	2.45E+06	VI
	mws0919	432.11	431.10	[M-H]-	285.00	Kaempferol-3-O-rhamnoside (Afzelin)(Kaempferin)	2.30E+06	2.38E+06	2.22E+06	2.38E+06	2.43E+06	VI
	pmp000117▲	432.14	433.15	[M+H]+	418.10	3,5,6,7,8,3',4'-Heptamethoxyflavone	5.17E+03	5.12E+03	4.09E+03	4.48E+03	4.40E+03	IV
	Lmfp004055▲	434.09	435.09	[M+H]+	303.05	Morin-3-O-xyloside*	1.44E+07	1.41E+07	1.43E+07	1.64E+07	1.57E+07	V
	mws2186	434.09	435.09	[M+H]+	303.00	Avicularin	9.67E+06	9.62E+06	9.29E+06	1.21E+07	9.29E+06	II

mws4183	434.09	433.08	[M-H]-	300.03	Quercetin-3-O-arabinoside(guaijaverin)*	9.40E+06	9.37E+06	9.62E+06	1.27E+07	1.06E+07	III
mws0045	448.10	449.11	[M+H]+	303.05	Quercetin-3-O-rhamnoside(Quercitrin)	1.97E+07	1.89E+07	1.96E+07	2.05E+07	2.11E+07	V
mws0913▲	448.10	447.09	[M-H]-	285.00	Kaempferol-3-O-galactoside (Trifolin)	7.82E+06	8.31E+06	7.74E+06	7.90E+06	8.45E+06	VI
pmn001640	450.08	449.07	[M-H]-	316.00	Myricetin-3-O-arabinoside	3.63E+04	5.08E+04	6.11E+04	9.54E+04	3.23E+04	I
Lmmn004625▲	450.12	449.11	[M-H]-	287.06	Dihydrokaempferol-7-O-glucoside*	9.69E+05	9.46E+05	8.14E+05	8.53E+05	6.51E+05	IV
Hmcp002539▲	462.12	463.12	[M+H]+	317.06	Isorhamnetin-3-O-rhamnoside	1.18E+06	1.26E+06	1.05E+06	1.13E+06	1.50E+06	VI
mws0061	464.10	463.09	[M-H]-	300.00	Quercetin-3-O-Galactoside (Hyperoside)	3.05E+06	3.26E+06	2.80E+06	2.92E+06	3.31E+06	VI
mws0091	464.10	463.09	[M-H]-	300.03	Quercetin-3-O-glucoside(Isoquercitrin)*	3.19E+06	3.16E+06	2.78E+06	2.97E+06	3.19E+06	V
pmp001309▲	464.10	465.10	[M+H]+	303.10	6-Hydroxykaempferol-7-O-glucoside	1.43E+06	1.45E+06	1.31E+06	1.50E+06	1.53E+06	V
pme1598▲	464.13	463.12	[M-H]-	301.00	Hesperetin-5-O-glucoside	7.81E+06	7.89E+06	7.38E+06	8.41E+06	8.16E+06	V
Hmjp003123▲	478.08	479.08	[M+H]+	303.06	Quercetin-5-O-glucuronide	3.60E+06	3.52E+06	3.94E+06	4.82E+06	3.01E+06	II
Lmyp004444▲	478.11	479.12	[M+H]+	317.06	Tricin-4'-methyl ether-3'-O-glucoside*	2.12E+06	2.29E+06	2.45E+06	2.78E+06	2.20E+06	VI
Hmcp002207▲	478.11	479.12	[M+H]+	317.06	Isorhamnetin-7-O-glucoside (Brassicin)	2.13E+06	2.21E+06	2.31E+06	2.71E+06	2.20E+06	III
Lmmn003398▲	490.11	489.11	[M-H]-	285.04	Kaempferol-3-O-(6''-acetyl)glucoside	2.04E+06	4.13E+06	5.58E+06	6.76E+06	3.18E+06	I
Lmmp003903▲	490.11	491.12	[M+H]+	287.05	Kaempferol-3-O-(2''-acetyl)glucoside*	3.88E+06	4.78E+06	5.26E+06	7.11E+06	2.54E+06	I
Hmln002199▲	506.11	505.10	[M-H]-	300.03	Quercetin-3-O-(6''-O-acetyl)-galactoside	2.34E+06	2.34E+06	2.78E+06	3.47E+06	3.15E+06	III
Lmmp003817▲	534.10	535.11	[M+H]+	287.05	Kaempferol-3-O-(6''-malonyl)glucoside	6.65E+06	9.86E+06	1.53E+07	1.82E+07	1.00E+07	I
pmp000589▲	550.10	551.10	[M+H]+	303.05	Quercetin-7-O-(6'-O-malonyl)-glucoside*	8.95E+06	8.69E+06	9.58E+06	1.07E+07	1.23E+07	V
Hmln002321▲	550.10	549.09	[M-H]-	300.00	Quercetin-3-O-(6''-O-malonyl)-glucoside	1.21E+04	1.13E+04	9.85E+03	8.35E+03	1.52E+04	III
pmb2979▲	550.13	549.12	[M-H]-	287.10	Hesperetin-7-O-(6''-malonylglucoside)	7.26E+04	9.96E+04	1.33E+05	1.38E+05	5.16E+04	I
pme2493▲	578.16	579.17	[M+H]+	147.00	Kaempferol-3,7-O-dirhamnoside (Kaempferitrin)	1.69E+05	1.91E+05	1.96E+05	1.90E+05	1.96E+05	VI
pmp001135▲	594.14	595.14	[M+H]+	285.00	Biondnoid I	4.95E+05	5.09E+05	6.09E+05	7.32E+05	3.92E+05	III
mws1290▲	594.14	593.13	[M-H]-	287.05	Tiliroside	1.23E+07	1.32E+07	1.70E+07	1.94E+07	1.03E+07	I
Lmjp00286▲7	594.16	595.17	[M+H]+	303.04	Kaempferol-3-O-neohesperidoside*	7.23E+06	6.99E+06	7.09E+06	9.96E+06	8.40E+06	III
Lmtp004044▲	596.14	597.14	[M+H]+	300.03	Quercetin-3-O-apiofuranosyl-(1→2)-galactoside	1.93E+05	2.70E+05	2.67E+05	2.61E+05	1.82E+05	II

	Lmln001951▲	596.14	595.13	[M-H]-	303.04	Quercetin-3-arabinosylglucoside*	1.07E+04	1.72E+04	1.44E+04	1.58E+04	7.49E+03	II
	Hmmp002336▲	610.15	611.16	[M+H]+	301.00	Quercetin-O-feruloyl-Pentoside	1.85E+07	1.93E+07	1.71E+07	1.83E+07	2.14E+07	VI
	mws0059	610.15	609.15	[M-H]-	300.00	Quercetin-3-O-rutinoside (Rutin)*	4.60E+05	5.35E+05	4.43E+05	4.39E+05	5.04E+05	VI
	pmn001583	610.15	609.15	[M-H]-	303.05	Quercetin-3-O-robinobioside	2.37E+06	2.66E+06	2.18E+06	2.13E+06	2.59E+06	VI
	Lmjp002461▲	610.15	611.16	[M+H]+	303.05	Quercetin-3-O-neohesperidoside	1.51E+07	1.58E+07	1.45E+07	1.51E+07	1.74E+07	VI
	Hmdp001872▲	616.11	617.11	[M+H]+	317.00	Quercetin-3-O-(2''-galloyl)-glucoside	1.71E+06	1.40E+06	1.28E+06	1.40E+06	1.43E+06	III
	pme1540▲	624.17	625.18	[M+H]+	300.00	Isorhamnetin-3-O-neohesperidoside	1.55E+06	1.67E+06	9.10E+05	3.91E+05	6.73E+05	V
	pmp000596▲	626.15	627.16	[M+H]+	465.10	Quercetin-3,7-O-diglucoside	1.99E+05	2.18E+05	1.63E+05	1.59E+05	1.95E+05	II
	pmp001311▲	626.15	627.16	[M+H]+	303.10	6-Hydroxykaempferol-7,6-O-Diglucoside	2.33E+06	2.59E+06	2.21E+06	2.46E+06	2.03E+06	I
	Lmtp003677▲	626.15	627.16	[M+H]+	303.04	Quercetin-3-O-galactoside-7-O-glucoside*	6.20E+04	8.12E+04	6.44E+04	6.97E+04	5.10E+04	II
	Hmcp001578▲	640.16	641.17	[M+H]+	479.11	Isorhamnetin-3,7-O-diglucoside*	3.57E+04	2.79E+04	2.77E+04	1.63E+04	1.78E+04	V
	Hmln001682▲	668.16	667.15	[M-H]-	462.08	Quercetin-3-O-(2''-acetyl)-glucosylgalactoside	2.45E+02	4.62E+03	3.37E+03	3.50E+03	1.60E+03	I
	Hmcp001629▲	696.15	697.16	[M+H]+	449.10	Kaempferol-3-O-(6''-Malonylglucoside)-7-O-Glucoside	3.88E+04	3.09E+05	8.25E+05	9.24E+05	7.47E+04	I
	pmb0709▲	712.15	713.16	[M+H]+	465.20	Quercetin-7-O-malonylglucosyl-glucoside	3.20E+03	1.59E+04	2.21E+04	3.44E+04	9.33E+03	I
	pmb0706▲	712.15	713.16	[M+H]+	465.10	Quercetin-5-O-malonylglucosyl-glucoside	1.95E+04	1.83E+05	1.91E+05	1.59E+05	6.82E+04	I
	Hmcp001757▲	756.21	757.22	[M+H]+	449.10	Quercetin-O-rhamnoside-O-glucoside-O-rhamnoside	1.68E+05	5.47E+05	4.90E+05	2.96E+05	1.99E+05	II
**Flavanols**	pme3285▲	274.08	275.09	[M+H]+	139.00	Afzelechin-3,5,7,4'-Tetrahydroxyflavan	1.65E+06	1.87E+06	1.15E+06	1.16E+06	1.28E+06	II
	mws1422▲	274.08	275.09	[M+H]+	107.00	Epiafzelechin*	4.09E+05	1.72E+06	1.53E+06	1.14E+06	3.29E+05	II
	pme0460	290.08	291.09	[M+H]+	139.04	Epicatechin*	3.11E+06	3.00E+06	2.78E+06	2.82E+06	2.70E+06	V
	mws0054	290.08	289.07	[M-H]-	245.00	Catechin	9.07E+05	8.74E+05	7.73E+05	7.33E+05	7.77E+05	IV
	mws0042▲	306.07	305.07	[M-H]-	125.00	Epigallocatechin*	2.88E+05	5.62E+05	6.46E+05	1.24E+06	3.17E+05	I
	mws0049▲	306.07	307.08	[M+H]+	139.00	Gallocatechin	1.43E+06	2.72E+06	2.81E+06	4.52E+06	1.82E+06	I
	mws0355▲	442.09	355.12	[M-H]-	169.02	Catechin gallate	3.04E+05	3.13E+05	2.27E+05	2.19E+05	2.63E+05	II

	pmn001415▲	452.11	451.10	[M-H]-	341.06	Catechin-(7,8-bc)-4β-(3,4-dihydroxyphenyl)-dihydro-2-(3H)-ne*	4.59E+04	4.31E+04	8.68E+04	2.69E+05	6.62E+04	VI
	pmn001416▲	452.11	451.10	[M-H]-	341.06	Catechin-(7,8-bc)-4α-(3,4-dihydroxyphenyl)-dihydro-2-(3H)-ne	4.78E+04	5.22E+04	8.85E+04	2.67E+05	7.03E+04	I
	HJN041▲	452.13	451.12	[M-H]-	289.07	Epicatechin glucoside	1.18E+07	1.32E+07	1.07E+07	1.00E+07	5.42E+06	II
	pmb3114▲	562.15	561.14	[M-H]-	289.20	Epicatechin-epiafzelechin	1.51E+06	1.60E+06	1.32E+06	1.22E+06	8.46E+05	V
	pmb2947▲	866.21	865.20	[M-H]-	407.10	Catechin-catechin-catechin	2.31E+05	2.70E+05	2.72E+05	2.37E+05	1.90E+05	I
**Isoflavones**	Lmgn002843▲	286.05	285.05	[M-H]-	199.04	2'-Hydroxyisoflavone	2.20E+03	4.33E+03	3.15E+03	5.13E+03	1.77E+03	I
	pmp000357▲	354.11	355.12	[M+H]+	299.05	Licoisoflavanone*	1.51E+05	9.96E+04	9.66E+04	8.65E+04	1.26E+05	III
	pmp000371▲	384.12	385.13	[M+H]+	329.06	Gancaonin D	1.08E+05	6.99E+04	6.15E+04	8.13E+04	1.01E+05	III
**Anthocyanin**	Smlp002532▲	419.10	419.10	[M]+	287.06	Cyanidin-3-O-arabinoside	9.49E+05	4.22E+06	5.60E+06	8.48E+06	9.25E+05	I
	pmb0550	449.11	449.11	[M]+	287.06	Cyanidin-3-O-glucoside (Kuromanin)	1.83E+05	3.21E+06	6.65E+06	9.26E+06	2.50E+05	I
	pme1398▲	465.10	465.10	[M]+	303.05	Delphinidin-3-O-glucoside (Mirtillin)	8.68E+05	7.16E+05	8.62E+05	1.12E+06	9.52E+05	III
	pmb0542▲	535.11	535.11	[M]+	287.06	Cyanidin-3-O-(6"-Malonylglucoside)	1.65E+04	2.78E+05	1.76E+06	3.14E+06	3.15E+04	I
	Smlp003276▲	579.13	579.13	[M]+	331.08	Malvidin-3-O-malonylglucoside	5.38E+04	4.76E+04	3.48E+04	2.63E+04	3.85E+04	V
	Smsp002643▲	579.17	579.17	[M]+	271.06	Pelargonidin-3-O-rutinoside	9.89E+06	9.00E+06	9.02E+06	8.10E+06	9.12E+06	III
	Lmjp001877▲	581.15	581.15	[M]+	287.05	Cyanidin 3-O-sambubioside [Cyanidin-3-O-(2"-O-β-xylosyl-β-glucoside)]	2.72E+05	2.59E+05	2.51E+05	2.29E+05	2.59E+05	V
	Lmpp003789▲	595.15	595.14	[M]+	287.06	Cyanidin-3-O-(6"-p-Coumaroylglucoside)	4.68E+06	4.84E+06	5.05E+06	7.05E+06	5.87E+06	VI
	pme1793	595.17	595.17	[M]+	271.06	Pelargonidin-3,5-diglucoside	6.06E+05	1.07E+06	4.92E+05	1.02E+06	3.06E+05	I

	Lmpp003662▲	611.14	611.14	[M]+	303.05	Delphinidin-3-O-(6"-p-Coumaroylglucoside)	1.72E+07	1.81E+07	1.59E+07	1.59E+07	1.98E+07	VI
	Lmpp003815▲	625.16	625.16	[M]+	317.07	Petunidin-3-(6"-p-Coumaroylglucoside)	1.79E+06	1.76E+06	1.02E+06	4.07E+05	8.61E+05	IV
	Lmjp001323▲	743.20	743.20	[M]+	287.06	Cyanidin 3-O-sambubioside-5-O-glucoside	1.21E+04	1.44E+04	1.19E+04	1.42E+04	5.90E+03	I
**Proanthocyanidins**	mws0024▲	170.02	169.01	[M-H]-	125.00	Gallic acid	1.16E+07	3.07E+07	1.58E+07	1.85E+07	1.62E+07	I
	pme0309▲	184.04	183.03	[M-H]-	124.00	3-O-Methylgallate	8.22E+06	6.61E+06	6.31E+06	5.85E+06	6.81E+06	III
	pme2246▲	302.01	301.00	[M-H]-	185.00	Ellagic acid	3.76E+06	4.18E+06	5.77E+06	6.18E+06	5.40E+06	I
	HmLn000873▲	332.08	331.07	[M-H]-	169.01	2-O-Galloyl-glucose*	1.56E+06	2.62E+06	3.96E+06	4.35E+06	1.58E+06	I
	HmLn000659▲	332.08	331.07	[M-H]-	169.01	3-O-Galloyl-glucose	2.09E+07	2.76E+07	2.59E+07	2.66E+07	1.95E+07	II
	pmn001519▲	336.05	335.04	[M-H]-	124.00	Galloyl Methyl gallate	3.03E+06	2.37E+06	1.79E+06	1.80E+06	1.51E+06	II
	Cmsn000894▲	362.08	361.08	[M-H]-	211.03	7-O-Galloyl-D-sedoheptulose	2.02E+04	3.08E+04	4.27E+04	6.03E+04	4.07E+04	I
	Hmln003405▲	446.05	447.06	[M-H]-	315.01	4-Rhamnosyl-ellagic acid	1.41E+06	1.70E+06	2.21E+06	2.05E+06	2.24E+06	VI
	Hmdn001667▲	464.06	463.05	[M-H]-	300.99	Ellagicacid-4-O-glucoside	3.34E+06	3.50E+06	4.43E+06	4.58E+06	2.98E+06	I
	Lmfn001258▲	468.09	467.08	[M-H]-	169.01	Maplexin D (2,4-Di-O-Galloyl-1,5-Anhydro-D-Glucitol)	3.78E+06	3.78E+06	3.77E+06	3.78E+06	4.39E+06	V
	Wmhn001495▲	484.08	483.08	[M-H]-	169.01	1,4-Di-O-galloyl-glcose	5.61E+06	7.54E+06	7.32E+06	5.99E+06	3.56E+06	II
	Lmfn000797▲	484.09	483.08	[M-H]-	169.01	2,3-Di-O-Galloyl-D-Glucose*	4.96E+06	5.49E+06	5.47E+06	6.67E+06	5.52E+06	VI
	Cmsn001563▲	514.10	513.09	[M-H]-	361.08	1,7-Di-O-galloyl-D-sedoheptulose	5.32E+03	4.84E+03	5.37E+03	4.71E+03	5.94E+03	III
	pme0432▲	576.13	575.12	[M-H]-	285.01	Procyanidin A2*	9.02E+03	1.87E+04	1.31E+04	1.34E+04	6.61E+03	II
	pmb0837▲	576.13	577.13	[M+H]+	425.30	Procyanidin A3*	1.47E+06	1.66E+06	1.49E+06	1.45E+06	1.28E+06	II
	pme0430▲	576.13	575.12	[M-H]-	285.00	Procyanidin A1	5.12E+03	1.05E+04	1.20E+04	1.86E+04	6.21E+03	I
	mws0836	578.14	577.14	[M-H]-	425.10	Procyanidin B1	1.41E+07	1.45E+07	1.38E+07	1.29E+07	1.15E+07	V
	pme0434▲	578.14	577.14	[M-H]-	407.10	Procyanidin B2*	1.69E+07	1.70E+07	1.68E+07	1.54E+07	1.36E+07	V
	pmn001667▲	578.14	577.14	[M-H]-	407.10	Procyanidin B4*	9.96E+03	1.03E+04	1.10E+04	2.32E+04	5.94E+03	I

pmp000274▲	578.14	579.15	[M+H]+	409.09	Procyanidin B3*	2.90E+06	2.67E+06	2.27E+06	1.93E+06	2.68E+06	II
pmp000275▲	580.16	581.17	[M+H]+	139.04	Gambiriin A1	2.55E+05	2.22E+05	2.22E+05	2.53E+05	4.35E+05	III
HJN074▲	592.16	591.15	[M-H]-	289.07	Procyanidin A6	1.63E+04	1.08E+04	1.62E+04	1.39E+04	2.25E+04	III
Cmhn001271▲	634.08	633.08	[M-H]-	463.05	Gemin D	8.22E+05	8.69E+05	1.00E+06	1.09E+06	9.96E+05	VI
Lmsn002110▲	634.08	633.07	[M-H]-	301.00	Corilagin	2.56E+05	2.98E+05	3.54E+05	4.25E+05	4.35E+05	VI
Lmfn001062▲	636.10	635.09	[M-H]-	169.01	2,4,6-Tri-O-galloyl-D-glucose	2.99E+05	3.73E+05	3.35E+05	3.38E+05	2.34E+05	II
Lmfn001209▲	636.10	635.09	[M-H]-	169.01	1,3,6-Tri-O-galloyl-D-glucose*	1.09E+05	1.99E+05	2.59E+05	1.98E+05	5.12E+04	I
Lmfn001337▲	636.10	635.09	[M-H]-	169.01	1,2,3-Tri-O-galloyl-D-glucose*	1.54E+05	1.89E+05	2.30E+05	2.36E+05	1.15E+05	II
Cmhn000855▲	650.08	649.07	[M-H]-	481.06	Valoneoyl-glucose	7.61E+05	1.06E+06	1.73E+06	2.40E+06	2.56E+06	VI
Cmhn001103▲	784.08	783.07	[M-H]-	481.06	Pedunculagin*	1.26E+06	1.46E+06	2.04E+06	2.16E+06	2.04E+06	I
Zmhp004555▲	784.08	785.08	[M+H]+	277.04	Casuariin*	5.63E+05	5.44E+05	5.94E+05	5.87E+05	8.61E+05	VI
Lmsn002071▲	786.09	785.08	[M-H]-	301.00	Tellimagrandin I*	2.37E+06	2.02E+06	2.09E+06	2.40E+06	1.91E+06	IV
HmdP001587▲	786.09	785.09	[M-H]-	301.00	Tercatain*	2.60E+06	2.34E+06	2.35E+06	2.74E+06	2.23E+06	III
pmp000280▲	866.21	867.21	[M+H]+	577.13	Procyanidin C2*	7.70E+06	7.72E+06	7.29E+06	6.88E+06	6.50E+06	V
pmn001646▲	866.21	865.20	[M-H]-	577.10	Procyanidin C1	9.16E+04	9.65E+04	9.45E+04	7.60E+04	6.36E+04	II
Cmhn002244▲	934.07	933.07	[M-H]-	631.06	Glansrin C*	7.63E+04	6.82E+04	9.98E+04	1.13E+05	9.02E+04	III
Cmhn002124▲	934.07	933.07	[M-H]-	631.06	Praecoxin D	7.03E+04	6.10E+04	9.89E+04	1.13E+05	8.90E+04	VI
Cmhn001611▲	936.09	935.09	[M-H]-	633.08	Casuarictin	5.69E+05	4.91E+05	6.59E+05	6.43E+05	7.73E+05	III
Hmln001664▲	952.08	951.08	[M-H]-	933.07	Geraniinic acid C	7.50E+06	8.62E+06	8.30E+06	8.01E+06	1.04E+07	VI
HJAP144▲	1018.22	1019.22	[M+H]+	731.16	Galloyl-procyanidin C2	1.04E+05	7.75E+04	6.33E+04	6.59E+04	5.34E+04	II
pmn001650▲	1154.27	1153.26	[M-H]-	575.10	Cinnamtannin B2*	6.79E+04	7.59E+04	9.06E+04	9.05E+04	5.69E+04	I
pmn001649▲	1154.27	1153.26	[M-H]-	1001.20	Cinnamtannin A2	2.25E+04	2.22E+04	2.98E+04	2.81E+04	1.64E+04	II

Note: a, theoretical; b, Experimental; MW, molecular weight; *, means there are several somerides of this compound; ▲means this compound was identified for the first time in rose.

Figure S1 The HPLC chromatograms of flavonoid metabolites from rose petal samples

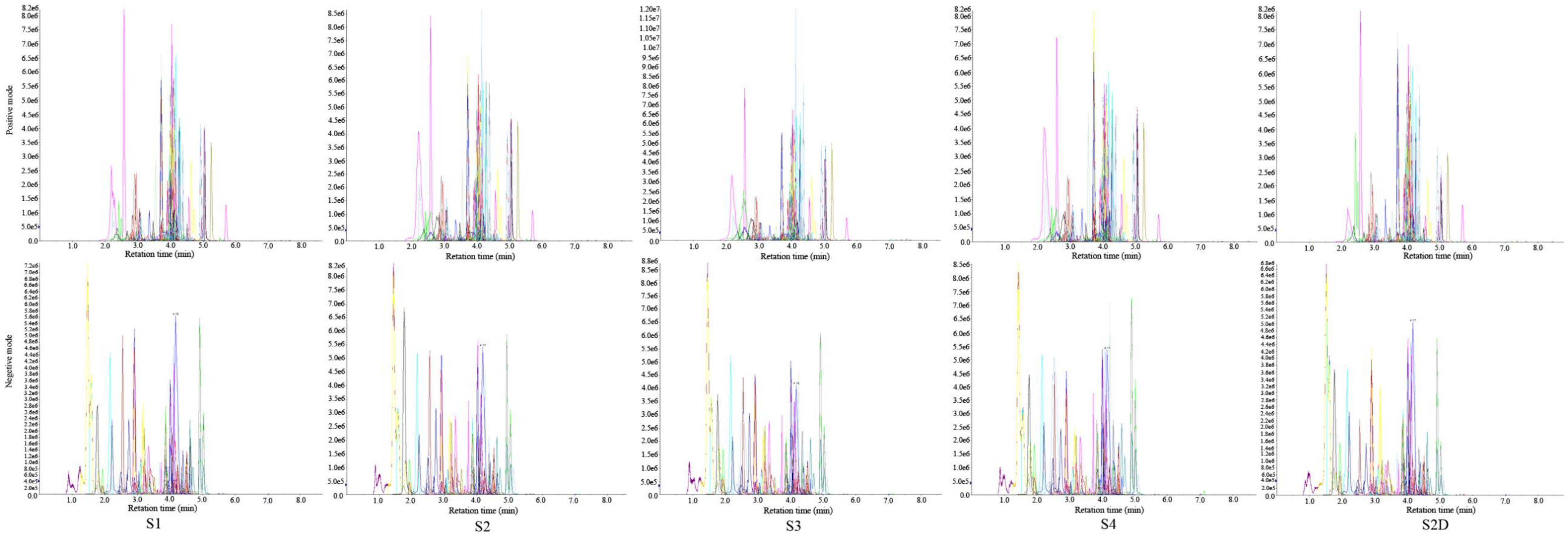

Supplement: Supplementary file 1 [file plants-10-02065-s001.zip › plants-1357514-supplementary.pdf]
